# The use of a contact force–sensing very-high-power short-duration radiofrequency catheter leads to significant efficiency improvement for dispersion-based ablation of persistent atrial fibrillation

**DOI:** 10.1016/j.hroo.2025.08.033

**Published:** 2025-08-30

**Authors:** Hind Regragui, Julien Seitz, Clément Bars, Edouard Gitenay, Anis Ayari, Jacques Monteau, Elisa Martinez, Andrea Ballatore, Jérôme Kalifa

**Affiliations:** 1 Hôpital Saint Joseph, Marseille, France; 2 Volta Medical, Marseille, France

**Keywords:** Atrial fibrillation, Catheter ablation, Personalized treatment, Artificial intelligence, Electrograms

**Key Findings**

▪ The use of a contact force–sensing very-high-power short-duration (vHPSD) radiofrequency catheter (aka, QDOT) during an artificial intelligence–guided personalized atrial fibrillation ablation allows for substantial gains in efficiency.
▪ Radiofrequency and fluoroscopy times were significantly reduced in the vHPSD cohort vs a non-vHPSD cohort.
▪ The use of a vHPSD radiofrequency catheter/QDOT is particularly adapted to extrapulmonary vein ablation workflows.

Pulmonary vein isolation (PVI) is the cornerstone for the ablation of persistent atrial fibrillation (AF). However, its results are often suboptimal. Recently, the TAILORED-AF trial showed that an artificial intelligence (AI)-guided patient-tailored approach targeting the spatiotemporal dispersion of electrograms (EGMs) is superior vs PVI-only procedures in patients with persistent and long-standing persistent AF.^1^ Spatiotemporal dispersion is defined as an ensemble of intracardiac EGMs forming a localized sequential activation in a distinct area, in which clusters of 3 or more adjacent bipolar EGMs show an intracardiac activation spanning the entire AF cycle length. In the TAILORED-AF trial, ablation was performed with radiofrequency (RF) energy according to conventional RF protocols described elsewhere.^1^ Despite promising outcomes, the main limitation of dispersion-based ablation is that it is associated with longer procedural, RF, and fluoroscopy times than a PVI-only approach. The QDOT MICRO catheter (Biosense Webster) integrates a very-high-power short-duration (vHPSD) RF technology. The use of this catheter represents an opportunity to reduce the procedural time of complex extra-PVI ablation procedures. Here, we evaluated the procedural efficiency of using a vHPSD catheter with AI-guided spatiotemporal dispersion mapping, a patient-tailored persistent AF ablation approach, which includes the ablation of extra-PV tissue.

We conducted a pilot single-center, nonrandomized investigation of patients with persistent AF undergoing first-time catheter ablation. All patients underwent a workflow similar to the one implemented in the TAILORED-AF trial, consisting of biatrial AI-guided spatiotemporal dispersion mapping followed by ablation of regions of interest.^1^ High-density biatrial mapping was conducted using a multielectrode multispline catheter (PENTARAY and OCTARAY, Biosense Webster), whereas the Volta AF-Xplorer decision support system (Volta Medical) was used to highlight dispersion regions in real time. Then, PVI and dispersion ablation were obtained with a vHPSD catheter following the protocol of “region-adapted maximal power,” that is, 90 W at the posterior wall and 50 W or 90 W at the anterior wall (vHPSD cohort).^2^ The study has been approved by Hôpital Saint Joseph’s institutional review board and adheres to the Declaration of Helsinki ethical guidelines. Patients provided a written informed consent.

Procedural characteristics and acute outcomes of the vHPSD cohort were compared with a propensity-matched cohort of eligible patients enrolled in the tailored arm of the TAILORED-AF trial. In that cohort, conventional RF catheters (ThermoCool SmartTouch, Biosense Webster; TactiCath, Abbott) were used following conventional ablation protocols^1^ (non-vHPSD cohort). The stabilized inverse probability of treatment weighting method was used to reduce selection bias by matching baseline characteristics (age, sex, weight, left atrial volume, AF maximum sustained duration, and AF type) between the vHPSD and the non-vHPSD cohorts.^3^

Baseline and procedural characteristics of the vHPSD (n = 54) and propensity score-matched non-vHPSD (n = 48) cohorts are presented in Table 1. The rates of acute AF termination by ablation and acute sinus rhythm conversion by ablation were similar (86% vs 87% [*P* = .90] and 68% vs 81% [*P* = .11] in the vHPSD and the non-vHPSD cohorts, respectively), whereas the procedure, RF, and fluoroscopy times were significantly reduced in the vHPSD cohort vs non-vHPSD cohort (128 ± 56 minutes vs 197 ± 50 minutes, *P* < .001; 26 ± 22 minutes vs 43 ± 12 minutes, *P* < .001; 3 ± 3 minutes vs 12 ± 25 minutes, *P* < .002). No complications were reported during procedures.

**Table 1:** Baseline and procedural characteristics

Propensity-matched stabilized IPTW method	vHPSD cohort	Non-vHPSD cohort	*P* value
	n = 54	n = 48	
Age (y)	71 ± 10	71 ± 9	.99
Sex, male	43 (78%)	40 (83%)	.48
Medical history			
Hypertension	31 (43%)	25 (51%)	.55
BMI (kg/m^2^)	28 ± 8	29 ± 5	.42
Diabetes	3 (5%)	14 (28%)	.002
Stroke	0 (0%)	1 (2%)	.24
Cardiac implantable device	4 (6%)	3 (6%)	.91
LVEF (%)	52 ± 19	51 ± 19	.31
NYHA functional class			<.001
Class I	3 (5%)	1 (3%)	
Class II	44 (82%)	21 (44%)	
Class III	7 (13%)	26 (53%)	
LA volume (mL)	183 ± 69	184 ± 47	.61
AF type			.89
Persistent AF	22 (41%)	19 (40%)	
Long-standing persistent AF	32 (59%)	29 (60%)	
AF history (y)	1.9 ± 2.9	3.8 ± 5.8	.002
AF maximum sustained duration (mo)	11 ± 13	12 ± 6	.28
AF maximum sustained duration category			.01
3–6 mo	16 (30%)	4 (8%)	
6–12 mo	10 (19%)	15 (32%)	
>12 mo	28 (51%)	29 (60%)	
Procedure time (min)	128 ± 56	197 ± 50	<.001
RF time (min)	26 ± 22	43 ± 12	<.001
Fluoroscopy time (min)	3 ± 3	12 ± 25	<.002
Acute AF termination by ablation	47 (86%)	42 (87%)	.90
Acute sinus rhythm conversion by ablation	37 (68%)	39 (81%)	.11

Values are given as n (%) or mean ± standard deviation. Continuous variables were analyzed by 2-sided Student *t* test or Wilcoxon test. Two-sided Fisher’s exact test was used for the categorical data.

AF = atrial fibrillation; BMI = body mass index; IPTW = inverse probability of treatment weighting; LA = left atrial; LVEF = left ventricular ejection fraction; NYHA = New York Heart Association; RF = radiofrequency; vHPSD = very-high-power short-duration.

Previously, the QDOT MICRO catheter has been demonstrated to reduce procedure and fluoroscopy times in patients with paroxysmal AF^4^ while retaining efficiency and improving safety.^2^ In particular, the 90 W ablations for a ≤4-second application protocol on the posterior wall are believed to be highly efficient, while remaining safe to use. In addition, the vHPSD catheter has been evaluated in patients with short-standing persistent AF (less than 4 months) and minimal extra-PVI linear ablation.^5^ Still, it was unclear whether vHPSD ablation is associated with gains in efficiency for *substantial extra-PV ablation strategies* and in patients with persistent AF for a year or more.

Overall, this single-center experience shows that the use of a vHPSD ablation strategy for extra-PVI procedures allows for a significant reduction in procedure, RF, and fluoroscopy times, while remaining safe to use. A 12-month follow-up will confirm whether the type of lesion generated by vHPSD ablation is sufficient to enable long-term freedom from AF/atrial tachycardia in patients with persistent and long-standing persistent AF.

## Disclosures

J.S. received speaker fees from Abbott, Biosense Webster, and Boston Scientific and reports being a cofounder and shareholder of Volta Medical. C.B. received consultant fees from Abbott and reports being a cofounder and shareholder of Volta Medical. J.K. reports being a cofounder and shareholder of Volta Medical. The other authors have nothing to declare.
